# *ASNS* can predict the poor prognosis of clear cell renal cell carcinoma

**DOI:** 10.3389/fonc.2022.882888

**Published:** 2022-08-16

**Authors:** Xinqiang Gan, Ruiji Liu, Hong Cheng, Weipu Mao, Ninghan Feng, Ming Chen

**Affiliations:** ^1^ Department of Urology, People’s Hospital of Putuo District, Shanghai, China; ^2^ Department of Urology, Affiliated Zhongda Hospital of Southeast University, Nanjing, China; ^3^ Department of Urology, Wuxi No.2 Hospital, Nanjing Medical University, Wuxi, China; ^4^ Nanjing Lishui District People’s Hospital, Zhongda Hospital Lishui Branch, Southeast University, Nanjing, China

**Keywords:** clear cell renal cell carcinoma, asparagine synthase, tumor microenvironment, immune infiltration, prognosis

## Abstract

**Purpose:**

Clear cell renal cell carcinoma (ccRCC) is one of the most common malignancies of the urinary system. This study was conducted to discover a new target that can predict the prognosis and promote the treatment of ccRCC.

**Methods:**

The raw data were downloaded from the TCGA database, and the predictive value of *ASNS* for various clinicopathological features was verified in the following analysis. Then, we analyzed the potential involvement of *ASNS* in tumor immunity and obtained the possible pathways involving *ASNS* through GO/KEGG enrichment analysis and GSEA. We also further verified our findings in pathological specimens of ccRCC patients.

**Results:**

*ASNS* expression was significantly increased in ccRCC, which was associated with advanced clinicopathological characteristics. It was an independent prognostic factor for overall survival in 535 patients with ccRCC. Immune cell infiltration analysis revealed that *ASNS* expression was related to T lymphocyte infiltration of tumors and poor prognosis. Moreover, we performed relevant functional enrichment analyses of *ASNS*.

**Conclusions:**

*ASNS* might play a significant role in the development and immune cell infiltration of ccRCC and serve as a valuable clinical prognostic biomarker.

## Introduction

Renal cell carcinoma (RCC) is a common cancer type, and approximately 430,000 new global cases and 170,000 RCC-related deaths occurred in 2020 (1). RCC accounts for approximately 3% of all cancers, with the highest incidence in Western countries, and 80%–90% of RCC are clear cell renal cell carcinoma (ccRCC) (2). None of the treatments were effective in patients with renal tumors subjected to the same surgical procedures and patients with advanced disease treated with similar drugs (3). Although an occasional response was reported, the available systemic therapies did not increase the survival of patients with advanced disease (2). Early detection and screening are priorities for RCC research (4). Therefore, discovering a new target of ccRCC, especially for those with advanced and metastatic diseases, is crucial.

Asparagine synthase (ASNS) catalyzes the synthesis of asparagine and glutamate from aspartic acid and glutamine in an ATP-dependent amidotransferase reaction, accompanied by glutamine deamidation (5). Large-scale loss-of-function analysis *in vitro* identified *ASNS* as cancer dependent in several solid malignancies; however, the specific mechanism has not been discovered (6). Knott et al. highlighted the role of ASNS in tumor growth and metastatic dissemination in a breast cancer model (7), prompting the necessity to evaluate the expression of *ASNS* and facilitating the prognosis of patients with ccRCC.

We downloaded raw data from The Cancer Genome Atlas (TCGA) database and verified the predictive value of *ASNS* for various clinicopathological features in the following analyses. We analyzed the potential involvement of ASNS in tumor immunity and identified the possible pathways associated with ASNS through Gene Ontology (GO)/Kyoto Encyclopedia of Genes and Genomes (KEGG) enrichment analyses and gene set enrichment analysis (GSEA). We further verified our findings in the pathological specimens of patients with ccRCC. To conclude, we found that *ASNS* was highly expressed in ccRCC patients, and its high expression could lead to a worse prognosis. Thus, our findings revealed that *ASNS* might play a significant role in the development and immune cell infiltration of ccRCC and serve as a valuable clinical prognostic biomarker.

## Materials and methods

### Public database collection

Gene expression (535 tumor and 72 normal samples), DNA methylation, phenotype, and survival data were downloaded from http://xena.ucsc.edu/. |logFC| > 1.5 and *p* < 0.01 were defined as the criteria for differentially expressed genes (DEGs), and DEGs were identified using the limma R package.

### COX univariate and multivariate analysis

Cox univariate and multivariate analyses were performed to determine the risk factors for ccRCC prognosis using R software.

### Survival analysis

Overall survival (OS) and progression-free survival rates of patients with ccRCC were investigated by Kaplan–Meier analysis.

### Correlation between immune infiltration and expression

TIMER2.0 (http://timer.comp-genomics.org/) was used for the comprehensive analysis of the relationship between *ASNS* expression and tumor-infiltrating immune cell levels, namely, CD4+ T cells, Tregs, CD8+ T cells, CTLA4, and PD-L1 (CD274) (8).

### Expression of hub genes and survival analysis

The STRING (https://string-db.org/) website analyzes protein–protein interactions using a unique set of computer prediction models (9). The expression levels of hub genes in tumor and normal tissues based on the ccRCC dataset were compared using the Wilcoxon rank-sum test (*p* < 0.05). OS analysis for the expression of hub genes between the high- and low-expression groups was performed, with a *p*-value of <0.05 indicating statistical significance.

### GEPIA2

GEPIA2 (http://gepia2.cancer-pku.cn/#index) uses standard processing procedures to analyze the data using many tumor samples and normal tissue samples (10). GEPIA2 analyzes the OS or disease-free survival of cancer patients based on gene expression. GEPIA2 uses the Log-rank test for hypothesis testing. The hazard ratio and 95% confidence interval are also included in survival plots.

### GO and KEGG enrichment analyses

According to *ASNS* expression level, ccRCC samples were sorted and divided into two groups of high and low expression, and the genes with differential expression were screened separately (|logFC| > 0.5, *p*-value < 0.05). The Clusterprofiler R package was used for GO enrichment analysis, which included molecular function, biological processes, and cellular components of DEGs, and KEGG enrichment analysis.

### Gene set enrichment analysis

The molecular mechanisms involved in ccRCC with different levels of *ASNS* expression were examined by the GSEA approach (11). The reference gene set used for GSEA was obtained from c7.immunesigdb.v7.5.1.symbols.gmt. Gene sets with a nominal *p*-value less than 0.05, |Normalized Enrichment Score| greater than 1, and false discovery rate less than 0.25 in the GSEA report were considered statistically significant.

### Western blotting

Western blotting was performed as described in our previous study (12).

### Immunohistochemistry

The expression and distribution of ASNS protein were detected by immunohistochemistry in paraffin-embedded tissue sections of each group. After routine paraffin dewaxing to water, antigen repair was performed. Hydrogen peroxide solution (3%) was added to remove endogenous catalase. Bovine serum albumin sealing solution (1%) was added, and the slides were incubated for 15 min. After the blocking solution was added, the primary antibody against ASNS (1:100 dilution) was added, and the slides were incubated overnight at 4°C. Thereafter, they were washed thrice with phosphate-buffered saline with Tween 20. The secondary antibody (1:100 dilution) was added, and the slides were incubated for 1 h at room temperature. DAB was added for color development, after which the slides were re-dyed with hematoxylin for 30 s. After washing with running water for 1 min, the slides were treated with 0.1% sodium bicarbonate to develop blue color. Then, the slides were subjected to dehydration and xylene treatment until the sections became transparent, mounted with neutral gum, observed under a microscope, and photographed.

The immunohistochemical staining intensity of ASNS was assessed using ImageJ software with the assistance of experienced pathologists in Affiliated Zhongda Hospital of Southeast University (13, 14).

### Sample information

Clinical data of 81 patients were collected at the Affiliated Zhongda Hospital of Southeast University from March 2019 to May 2022. All patients were diagnosed with ccRCC (unilateral) and had no other carcinomas. All patients did not undergo any preoperative therapy for the carcinoma (chemotherapy, targeted therapy, immunotherapy, etc.). Age distribution: 19–85 years old. Surgical modalities: Partial nephrectomy or radical nephrectomy. Neoplasm histological grade criteria: Fuhrman nuclear grading system. Pathological stage criteria: American Joint Committee on Cancer (AJCC).

### Statistical analysis

Analyses were performed using IBM SPSS for MAC version 26.0. The Mann–Whitney test was used to compare continuous variables between the two groups. The immunohistochemical staining intensity and clinical characteristics, such as gender, age, location, tumor size, TNM stage, AJCC stage, and grade were subjected to Spearman rank correlation analysis. *p*-values less than 0.05 were considered statistically significant.

## Results

### Overexpression of ASNS mRNA in ccRCC is related to poor prognosis

**Figure 1:**
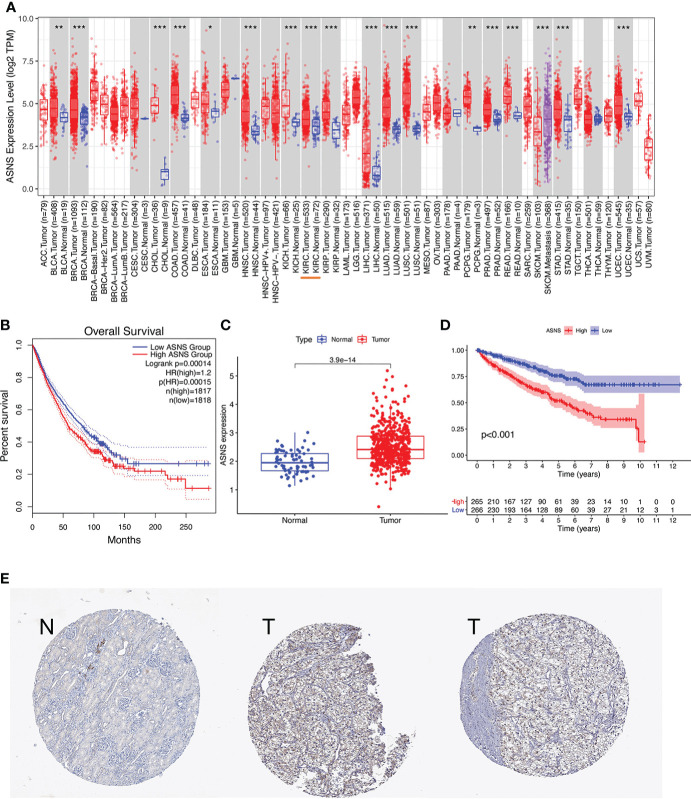
Expression of *ASNS*. **(A)** Pan-cancer expression of *ASNS*. **(B)** Prognostic values of *ASNS* gene in multiple cancers (BRCA, CESC, CHOL, ESCA, KIRC, LIHC, LUAD, LUSC, PAAD, and STAD). **(C)** *ASNS* expression in ccRCC and normal tissues. **(D)** Relationship between *ASNS* expression levels and prognosis, i.e., overall survival (OS). **(E)** ASNS protein level in ccRCC from the HPA database. The statistical significance computed by the Wilcoxon test is annotated by the number of stars (*: p-value <0.05; **: p-value <0.01; ***: p-value <0.001).

The expression pattern of *ASNS* in multiple pan-cancers was evaluated by using data from TIMER2.0, and the prognostic values of *ASNS* in multiple cancers (invasive breast carcinoma, cervical squamous cell carcinoma, endocervical adenocarcinoma, cholangiocarcinoma, esophageal carcinoma, kidney renal clear cell carcinoma, hepatocellular carcinoma, lung adenocarcinoma, lung squamous cell carcinoma, pancreatic adenocarcinoma, and stomach adenocarcinoma) are shown in the survival map prepared using GEPIA2 (**Figures 1A, B**). The pan-cancer overexpression of *ASNS* was linked to poor prognosis. The mRNA data of ccRCC were downloaded from TCGA (https://xenabrowser.net/datapages/) and included 535 tumor tissues and 72 normal tissues. *ASNS* was highly expressed in ccRCC compared with normal renal tissues (*p* < 0.001; **Figure 1C**) and was correlated with poor prognosis, i.e., OS (*p* < 0.001; **Figure 1D**). Furthermore, the high protein expression level of *ASNS* in ccRCC was verified by data from the Human Protein Atlas (HPA) databases (https://www.proteinatlas.org/) (**Figure 1E**).

**Figure 2:**
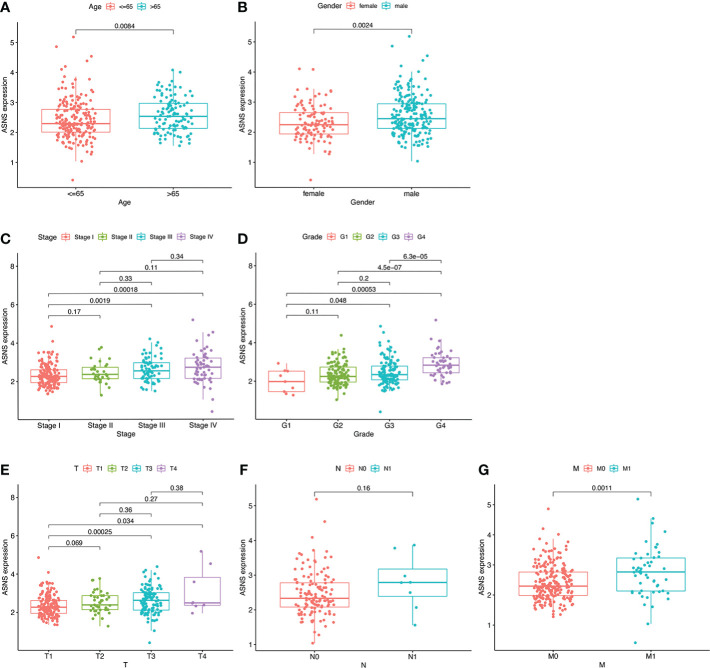
Expression pattern of *ASNS* in relation to several clinicopathological features. **(A, B)** *ASNS* expression in samples from patients stratified by age and gender. **(C–G)** *ASNS* expression in samples stratified by pathological stage, neoplasm histological grade, T stage, N stage, and M stage, respectively.

For further clinical research, the expression pattern of *ASNS* was studied in relation to several clinicopathological features, and the data demonstrated that *ASNS* expression gradually increased with tumor progression (**Figure 2**). Therefore, *ASNS* could be an independent factor predicting ccRCC prognosis.

### *ASNS* is an independent prognostic factor for ccRCC

**Figure 3:**
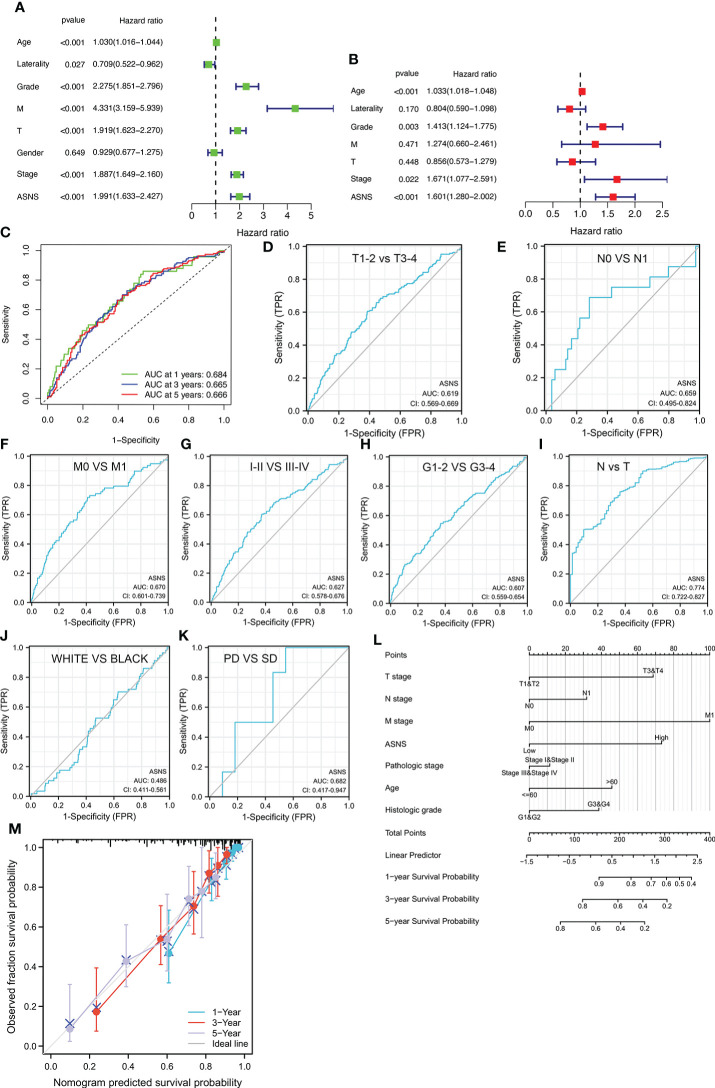
Identification of *ASNS* as an independent prognostic factor for ccRCC. **(A, B)** Univariate and multivariate independent prognostic analysis of *ASNS*. **(C)** Receiver operating characteristic (ROC) curve predicting the correlation between *ASNS* expression and OS. **(D–K)** ROC curve predicting the correlation between *ASNS* expression and clinicopathological features [T1–2 *vs*. T3–4, N0 *vs*. N1, M0 *vs*. M1, pathological stage I–II *vs*. III–IV, neoplasm histological grade 1–2 *vs*. 3–4, normal *vs*. tumor, race (white *vs*. black), and primary therapeutic outcome, i.e., partial disease *vs*. stable disease]. **(L, M)** Nomogram and calibration plots, respectively.

Univariate independent prognostic analysis demonstrated that neoplasm histologic grades, pathological T&M stages, tumor stages, and the expression of *ASNS* were significant factors that could predict ccRCC prognosis (*p* < 0.001; hazard ratio [HR] > 1; **Figure 3A**). Meanwhile, multivariate prognostic analysis showed that *ASNS* could be an independent prognostic factor for ccRCC (*p* < 0.001; HR > 1; **Figure 3B**). Moreover, the receiver operating characteristic (ROC) curve was used to analyze the accuracy of *ASNS* to predict the survival of ccRCC. *ASNS* predicted the survival period of ccRCC patients at 1, 3, and 5 years, which was significant (area under the curve [AUC] > 0.6; **Figure 3C**). Subsequently, serial ROC analysis showed favorable diagnostic values for *ASNS* to predict various clinicopathological features (**Figures 3D–K**). Ultimately, the above results were validated using nomogram and calibration plots, which showed that *ASNS* had excellent potential for predicting clinicopathological features in ccRCC (**Figures 3L, M**).

### DNA methylation analysis of *ASNS* gene

**Figure 4:**
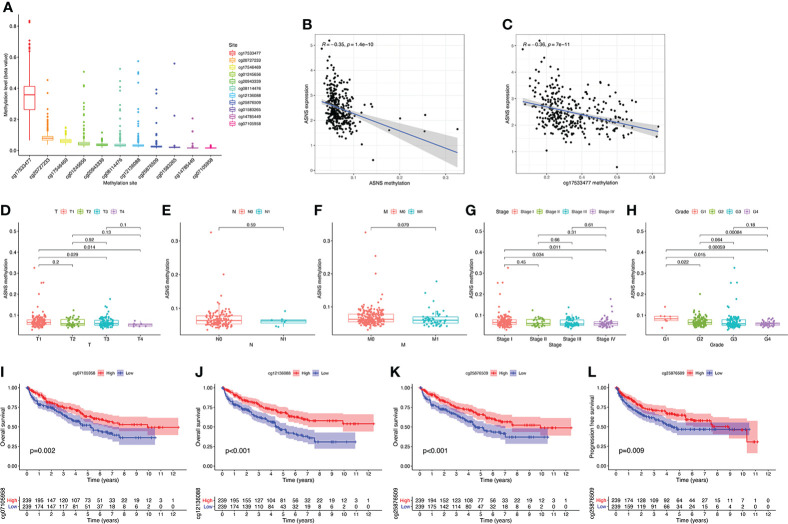
DNA methylation analysis of *ASNS*. **(A)** Methylation levels at different sites of *ASNS*. **(B, C)** The relationship between *ASNS* expression level and methylation level. **(D–H)** *ASNS* methylation in samples stratified by T stage, N stage, M stage, neoplasm histological grade, and pathological stage. **(I–L)** Methylation levels of *ASNS* on the prognosis of ccRCC (OS and progression-free survival rates).

To further explore the regulation of *ASNS* in ccRCC, we performed a series of methylation analyses. Methylation levels were detected at the promoter region sites of the *ASNS* gene (**Figure 4A**), and *ASNS* expression was inversely proportional to the methylation level (**Figures 4B, C**). We further determined the relationship between *ASNS* gene methylation level and various clinicopathological features such as pathologic TNM stages (**Figures 4D–F**), tumor stages (**Figure 4G**), and neoplasm histologic grades (**Figure 4H**) of ccRCC. The results demonstrated that the methylation level of *ASNS* gradually decreased with tumor progression. Furthermore, the lower methylation level of *ASNS* led to a worse prognosis, such as poorer OS rates (*p* < 0.001; **Figures 4I–K**) and poorer progression-free survival (*p* < 0.001; **Figure 4L**). The decrease in the methylation level of *ASNS* with tumor progression indicates that the methylation modification of *ASNS* plays a key regulatory role in the expression of *ASNS*.

### Immune infiltration analysis of *ASNS* in ccRCC

**Figure 5:**
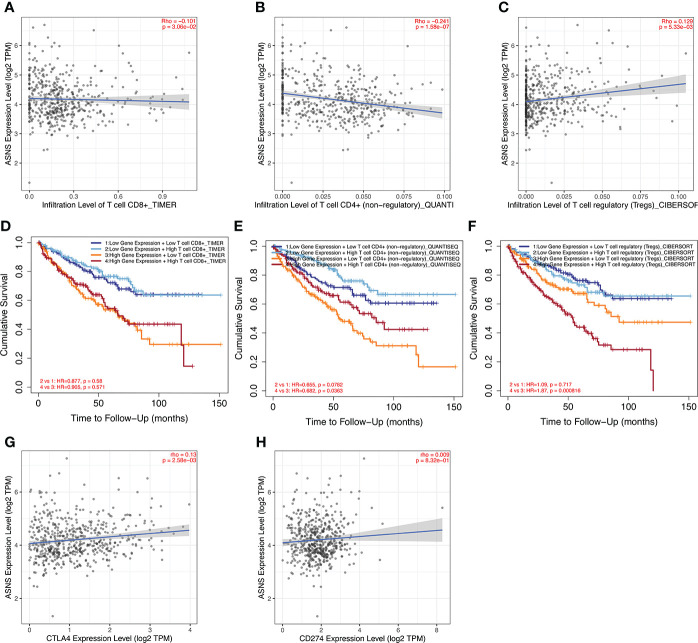
Immune infiltration analysis of *ASNS* in ccRCC. **(A–C)** Correlation between *ASNS* expression and immune cell infiltration in ccRCC. **(D–F)** Correlation between immune cell infiltration and prognosis. **(G, H)** Correlation between *ASNS* and expression level of immune checkpoint molecules.

ccRCC has a relatively high stromal score and immune score among common cancers (15). High immune scores and infiltration of Tregs are significantly associated with poor OS, high tumor stage, and more chances of metastases in ccRCC (16). To further investigate the relationship between *ASNS* expression and the immune microenvironment in ccRCC, we performed immune infiltration analysis using TIMER2.0. The immune infiltration level of non-regulatory CD^4+^ T cells was negatively correlated with *ASNS* expression (**Figure 5B**), whereas Tregs were positively correlated (**Figure 5C**). Subsequent analyses showed that higher CD^4+^ T-cell infiltration was associated with better prognosis (**Figure 5E**), and higher Tregs infiltration was associated with poorer prognosis (**Figure 5F**). The correlation between CD^8+^ T-cell infiltration and *ASNS* was also analyzed; however, the results were insignificant (**Figures 5A, D**). Finally, *ASNS* expression correlated positively with the expression of immune checkpoints such as CTLA4, and PD-L1 (CD274) was positive (**Figures 5G, H**).

### Cell function and pathway analysis in *ASNS*

**Figure 6:**
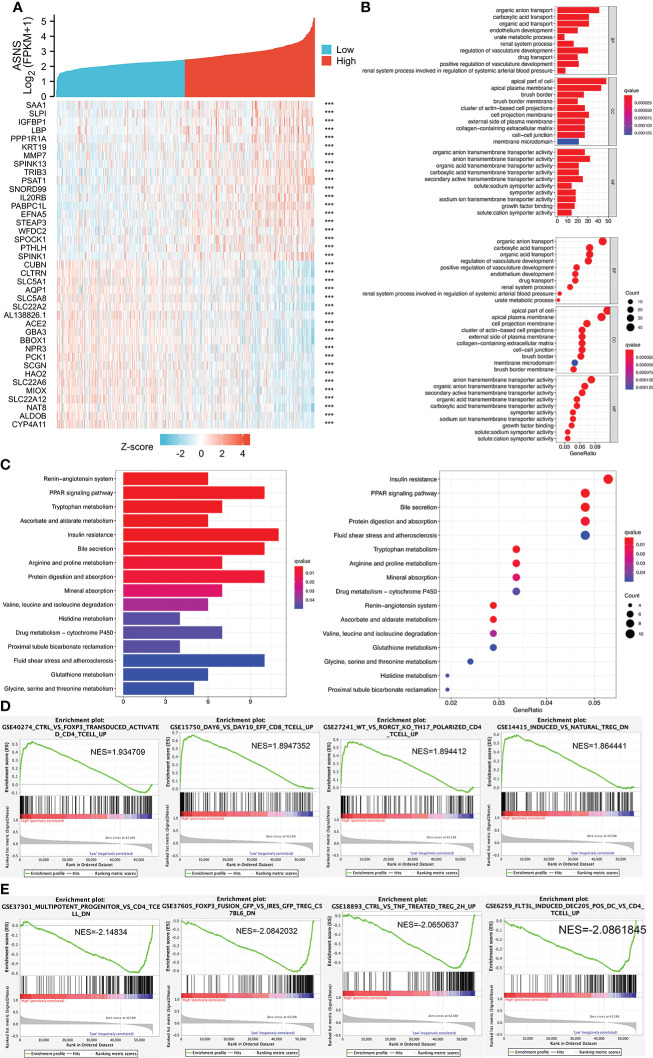
Cell function and pathway analysis involved in *ASNS*. **(A)** Heatmap of the expression patterns of the top 20 positively and negatively correlated genes of *ASNS* in ccRCC. **(B)** GO enrichment analysis. **(C)** KEGG enrichment analysis. **(D, E)** Gene set enrichment analysis (GSEA).

We analyzed the positively and negatively correlated genes of *ASNS* in ccRCC and plotted heatmaps to show the expression patterns of the top 20 *ASNS*-associated genes (**Figure 6A**). To further determine the functions of *ASNS* in tumor progression and prognosis, GO and KEGG enrichment analyses were performed. GO enrichment analysis found that ASNS protein may be located on the cell membrane and regulate the molecular transport in cells (***q***-value <0.01; **Figure 6B**). KEGG enrichment analysis indicated that *ASNS* might participate in the regulation of insulin resistance, PPAR signaling pathway, and amino acid metabolism (***q***-value <0.05; **Figure 6C**). Subsequent GSEA suggested that *ASNS* might be involved in CD 4+ T-cell infiltration-related signaling in ccRCC (**Figures 6D, E**), which is also consistent with the previous analysis results.

### Protein–protein interaction network of *ASNS*

**Figure 7:**
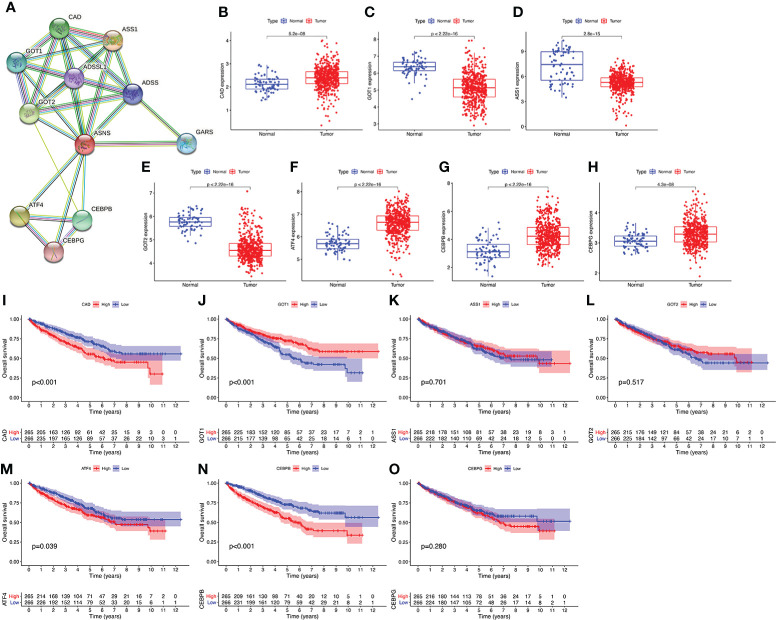
Protein–protein interaction (PPI) network analysis of ASNS. **(A)** The top 10 hub genes of the PPI network. **(B–O)** The expression pattern and prognostic values of these hub genes in ccRCC (the three missing genes were not found in the TCGA-KIRC database).

We performed a PPI network analysis of ASNS using the STRING website to explore potential interactions between ASNS and other proteins. The top 10 hub genes were selected from the PPI network, and the expression patterns and prognostic values in ccRCC were studied (**Figure 7**).

### Validation of *ASNS* in cells and pathological tissues of ccRCC

**Figure 8:**
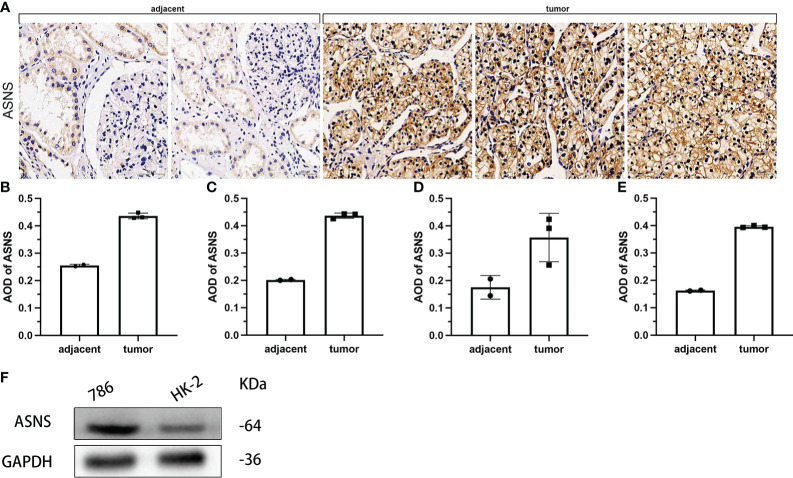
Validation of ASNS in cells and pathological tissues of ccRCC. **(A)** Results of immunohistochemical analysis of ASNS in ccRCC tissues and adjacent normal tissues. **(B–E)** Quantitative immunohistochemical analysis results of ASNS in four samples. **(F)** Western blot result of ASNS in 786-O and HK-2 cells.

To further verify the effect of *ASNS* on ccRCC, we evaluated the expression of *ASNS* in ccRCC tissues and human ccRCC cell lines. Compared with adjacent normal renal tissues, *ASNS* was highly expressed in ccRCC tissues (**Figure 8A**). ImageJ software was used to quantitatively analyze the expression of *ASNS* (**Figures 8B–E**). Finally, we evaluated the expression of ASNS protein in 786-O cells (human ccRCC) and HK-2 cells (Human Kidney-2 cells), and the results were significant (**Figure 8F**).

In addition, the above results were further validated using the data from our cohort. It was further verified that *ASNS* was highly expressed in ccRCC tissues compared with adjacent tissues (*p* < 0.01, **Table 1**). Moreover, high ASNS expression was not correlated with age, gender, and tumor location (*p* > 0.05, **Table 2**), but was significantly positively associated with tumor size, T stage, the American Joint Committee on Cancer (AJCC) status, and neoplasm histological grade (*p* < 0.001, **Table 2**). These were also consistent with our previous results.

**Table 1:** AOD of ASNS in tumor tissues compared with normal tissues.

Group	*n*	AOD of ASNS (mean)	*p*-value
Adjacent tissues	81	0.206	<0.01
Tumor tissues	81	0.444	

**Table 2:** Baseline characteristics of ccRCC patients in our cohort.

Characteristic	ASNS level		*p*-value
	Low	High	
*n*	40	41	
Gender, *n* (%)			1.000
Female	13 (16%)	13 (16%)	
Male	27 (33.3%)	28 (34.6%)	
Age (years), *n* (%)			0.429
<60	24 (29.6%)	20 (24.7%)	
≥60	16 (19.8%)	21 (25.9%)	
Location, *n* (%)			0.891
Left	25 (30.9%)	24 (29.6%)	
Right	15 (18.5%)	17 (21%)	
Tumor size, *n* (%)			< 0.001
<5 cm	32 (39.5%)	13 (16%)	
≥5 cm	8 (9.9%)	28 (34.6%)	
T stage, *n* (%)			< 0.001
T1	37 (45.7%)	12 (14.8%)	
T2	2 (2.5%)	8 (9.9%)	
T3	1 (1.2%)	19 (23.5%)	
T4	0 (0%)	2 (2.5%)	
N stage, *n* (%)			1.000
N0	40 (49.4%)	40 (49.4%)	
N1	0 (0%)	1 (1.2%)	
M stage, *n* (%)			0.116
M0	40 (49.4%)	37 (45.7%)	
M1	0 (0%)	4 (4.9%)	
AJCC stage, *n* (%)			< 0.001
I	37 (45.7%)	11 (13.6%)	
II	2 (2.5%)	8 (9.9%)	
III	1 (1.2%)	17 (21%)	
IV	0 (0%)	5 (6.2%)	
Grade, *n* (%)			< 0.001
1	12 (14.8%)	1 (1.2%)	
2	15 (18.5%)	9 (11.1%)	
3	13 (16%)	22 (27.2%)	
4	0 (0%)	9 (11.1%)	

## Discussion

ccRCC is one of the most common malignancies of the urinary system. Several biomarkers such as ciRS-7 (17), CA9 (18), Ki-67 (19), Bcl-2 (20), and PTEN (21) can predict ccRCC prognosis. We aimed to identify a novel biomarker to predict the prognosis of ccRCC. We found that *ASNS* might play a significant role in the development of ccRCC and serve as a valuable clinical prognostic biomarker of ccRCC.

The upregulation of *ASNS* expression responds to single or combined restrictions on many amino acids, including the most essential amino acids (22). Amino acid starvation-induced upregulation of *ASNS* is mediated by activating transcription factor 4 (ATF4). ASNS is the transcriptional target of ATF4, responding to amino acid starvation *via* the GCN2/eIF2α axis. The GCN2/eIF2α/ATF4 pathway is activated in primary solid tumors, indicating that the regulation of asparagine production in a nutrient-limited environment is essential for the progression of solid tumors (23). Maintaining intracellular asparagine levels is necessary for cancer cell growth (24). Asparagine is an important regulator of cancer cell amino acid homeostasis, anabolic metabolism, and proliferation (24). Therefore, we suspected that *ASNS* might play an important role in regulating the intracellular and extracellular metabolism of amino acids, thereby promoting the development of ccRCC, consistent with our results (**Figure 6**).

*ASNS* knockdown leads to cell death even in the presence of glutamine, which can be reversed by adding exogenous asparagine (25). The standard treatment for childhood acute lymphoblastic leukemia includes the infusion of bacterial ASNase as a principal component of combination chemotherapy (26). Circulating ASNase causes the rapid consumption of plasma asparagine and depletion of intracellular asparagine, starving leukemia cells and preventing their further growth (27). Hence, the growth of solid tumors might be regulated by ASNS protein levels. A study showed that in about 70% of human pancreatic ductal cancer samples, the level of ASNS protein was below the detection level, which indicates that some pancreatic tumors may be sensitive to ASNase treatment (28). In another study using ovarian cell lines, a negative correlation was observed between ASNase treatment efficacy and ASNS protein levels rather than ASNS mRNA levels (29). Pancreatic cancer cells overexpressing ASNS exhibit increased resistance to apoptosis induced by cis-diamine-dichloro platinum, which is related to the inhibition of JUN NH2-terminal kinase activation by ASNS (30). Therefore, whether ASNase treatment can inhibit tumor cells in ccRCC needs to be researched.

As shown by immunohistochemistry of human pancreatic tissues, pancreatic ASNS protein expression was largely correlated with exocrine cells (28). The ASNS protein is released from primary mouse tumors into the serum at a rate proportional to tumor growth; therefore, serum ASNS activity may be a valuable marker for the lysis of pancreatic exocrine cells (31). The secretion pattern of ASNS protein needs to be investigated in ccRCC.

Several studies on asparagine-dependent and asparagine-independent cell lines revealed the correlation between DNA methylation in the *ASNS* locus and *ASNS* expression (32, 33). High *ASNS* promoter methylation is associated with low *ASNS* expression, and 5-Aza-dC treatment enhances *ASNS* expression (34). In acute lymphoblastic leukemia bone marrow samples, most B cells and T cells showed methylation of the *ASNS* promoter, in contrast to the lack of methylation observed in brain and breast tumors (35). Akagi et al. hypothesized that *ASNS* methylation might be the basis for the susceptibility of acute lymphoblastic leukemia cells to ASNase chemotherapy (35). We found that the cg17533477 site of *ASNS* had high-level methylation in ccRCC (**Figure 4A**), indicating that ccRCC is more sensitive to ASNase chemotherapy. Moreover, the higher methylation level of the *ASNS* gene leads to a better prognosis (**Figures 4I–L**).

Multiple studies have shown that tumor-infiltrating immune cells regulate cancer progression and promote tumor development (36, 37). CD^4+^ helper T cells and cytotoxic CD^8+^ T cells play an important role in tumor prevention by targeting antigenic tumor cells, and CD^8+^ T cells are associated with better clinical outcomes and response to immunotherapy in many cancers (38–40). T cells (CD^4+^ T cells and CD^8+^ T cells) are the primary type of immune cells in ccRCC tumors (41). Effector T cells and mature dendritic cells may contribute to antitumor immune responses, whereas Tregs have the opposite effect (42), consistent with our findings (**Figures 5B, C, E, F**).

**Figure 9:**
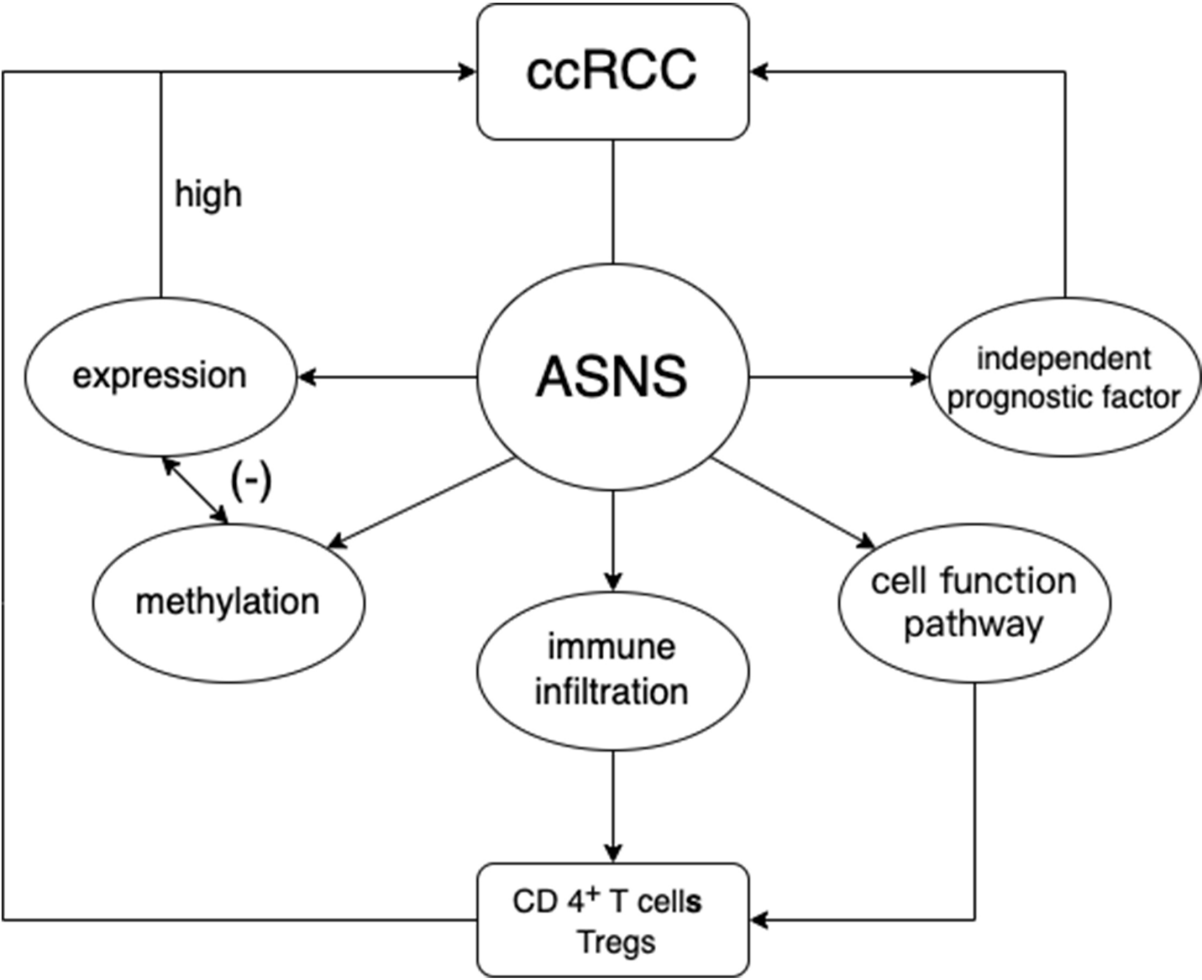
Study flowchart.

In our study, we analyzed the significant role of *ASNS* in the development of ccRCC and verified the possibility and feasibility of using it as an independent prognostic factor for ccRCC prognosis (**Figure 9**). Taken together, *ASNS* could act as an independent prognostic factor for ccRCC and might play a crucial role in tumor progression and immune cell infiltration. However, robust experimental data are not available to confirm our findings. Only preliminary analyses showed the function of *ASNS* in ccRCC, but certain signaling pathways in which it plays an important role remain to be studied. Due to the small number of data included in this center (only 81 cases), our validation results did not show that ASNS expression levels were associated with lymph node metastases and distant metastases. To further explore the vital role of *ASNS* in ccRCC, more detailed basic experimental research and clinical studies are required.

## Data availability statement

The datasets presented in this study can be found in online repositories. The names of the repository/repositories and accession number(s) can be found below: https://xenabrowser.net/datapages/.

## Ethics statement

The studies involving human participants were reviewed and approved by Ethics Committee and Institutional Review Board for Clinical Research of Zhongda Hospital (ZDKYSB077) (43, 44, 45, 46). This study is a retrospective study that only collected clinical data from patients, and the ethical committee approved the exemption of informed consent.

## Author contributions

XG, RL, MC, WM, NF, and HC designed the study. XG and RL conducted the study, maintained the data, analyzed the data, and prepared the figures. XG, RL, WM, and NF reviewed and revised the manuscript. All authors contributed to the article and approved the submitted version.

## Funding

This study was funded by The National Natural Science Foundation of China (No. 81370849, 81300472, 81070592, 81202268, 81202034); Natural Science Foundation of Jiangsu Province (BK20161434, BL2013032, BK20150642 and BK2012336). Major Project of Jiangsu Commission of Health: (No: ZD2021002); Wuxi “Taihu Talents Program” Medical Expert Team Project (No: THRCJH20200901, THRCJH20200902).

## Acknowledgments

We thank Bullet Edits Limited for the linguistic editing and proofreading of the manuscript.

## Conflict of interest

The authors declare that the research was conducted in the absence of any commercial or financial relationships that could be construed as a potential conflict of interest.

## Publisher’s note

All claims expressed in this article are solely those of the authors and do not necessarily represent those of their affiliated organizations, or those of the publisher, the editors and the reviewers. Any product that may be evaluated in this article, or claim that may be made by its manufacturer, is not guaranteed or endorsed by the publisher.
